# Mevacor/Poly(vinyl acetate/2-hydroxyethyl methacrylate) as Solid Solution: Preparation, Solubility Enhancement and Drug Delivery

**DOI:** 10.3390/polym15193927

**Published:** 2023-09-28

**Authors:** Mohammed Alassaf, Saad Mohammed Alqahtani, Rana Salem Al Khulaifi, Waseem Sharaf Saeed, Faisal S. Alsubaie, Abdelhabib Semlali, Taieb Aouak

**Affiliations:** 1 Department of Chemistry, College of Science, King Saud University, Riyadh 11451, Saudi Arabia; alassafm926@gmail.com (M.A.); salqahtani2@ksu.edu.sa (S.M.A.); ranaalkhulaifi@gmail.com (R.S.A.K.); wsaeed@ksu.edu.sa (W.S.S.);; 2 Groupe de Recherche en Écologie Buccale, Faculté de Médecine Dentaire, Université Laval, Quebec City, QC G1V 0A6, Canada

**Keywords:** Mevacor/poly(vinyl acetate-co-2-hydroxyl methyl methacrylate), solid solution, photo-copolymerization, LED light, Mevacor delivery, solubility enhancement

## Abstract

Mevacor/Poly(vinyl acetate-co-2-hydroxyethyl methacrylate) drug carrier systems (MVR/VAC-HEMA) containing different Mevacor (MVR) contents were prepared in one pot by free radical copolymerization of vinyl acetate with 2-hydroxyethyl methacrylate using an LED lamp light in the presence of camphorquinone as a photoinitiator and Mevacor as a drug filler. The prepared material was characterized by FTIR, ^1^H NMR, DSC, SEM and XRD methods. Different parameters influencing the efficiency in the Mecvacor-water solubility and the drug delivery of this system, such as the swelling capacity of the carrier, the amount of Mevacor loaded and the pH medium have been widely investigated. The results obtained revealed that the Mevacor particles were uniformly dispersed in their molecular state in the copolymer matrix forming a solid solution; the cell toxicity of the virgin poly(vinyl acetate-co-2-hydroxy ethyl methacrylate) (VAC-HEMA) and MVR/VAC-HEMA drug carrier system exhibited no significant effect on their viability when between 0.25 and 2.00 wt% was loaded in these materials; the average swelling capacity of VAC-HEMA material in water was found to be 45.16 wt%, which was practically unaffected by the pH medium and the solubility of MVR deduced from the release process reached more than 22 and 37 times that of the powder dissolved directly in pH 1 and 7 media, respectively. The in vitro MVR release kinetic study revealed that the MVR/VAC-HEMA system containing 0.5 wt% MVR exhibited the best performance in the short gastrointestinal transit (GITT), while that containing 2.0 wt% is for the long transit as they were able to considerably reduce the minimum release of this drug in the stomach (pH1).

## 1. Introduction

Today, polymer materials occupy practically all sectors of industry. In the biomedical field, these materials are now involved in several formulations, particularly in the drug delivery domain in order to obtain desirable pharmacokinetics and therapeutic indices. Controlling the amount of drug released over time and its rate are key elements in the development of drug delivery systems—the rapid release of medication can have adverse effects on the body, and too little can prevent this drug from achieving the desired effectiveness. Therefore, drug carriers must be designed in a fashion that allows them to deliver the optimal dosage for the optimal duration of time in order to render drugs more effective [1,2,3].

Hydrogel-based drug delivery systems provide an alternative to the conventional drug formulations. They have been utilized in recent years to tackle the inadequate bioavailability of drugs. Hydrogels are highly water-absorbent mesh networks that are formed naturally or synthetically. The chains of polymers in these systems are crosslinked physically by means of many intermolecular forces (e.g., hydrogen bonding, dispersion interaction) or bound to each other covalently [4]. In many studies, researchers made use of polymer hydrogels as carriers due to their biocompatibility and physicochemical tolerability [5,6]. Among these studies, several investigations have been conducted in which polymer hydrogels were employed to enhance bioavailability and controlled the amount of drugs being released.

**Scheme 1:**
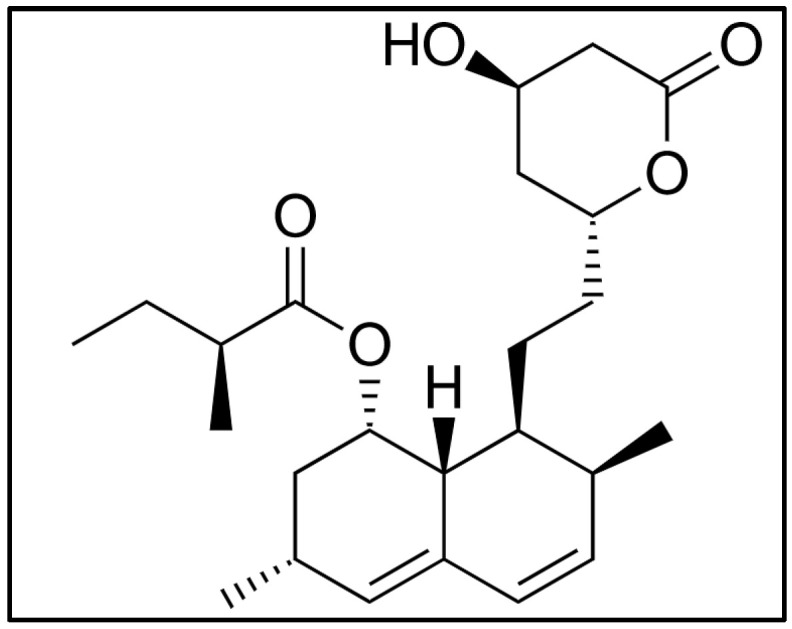
Chemical structure of Mevacor (MVR).

Mevacor (MVR) (Scheme 1), sold under the brand name “Lovastatin” or “Altoprev” among others, belongs to a class of drugs called HMG-CoA reductase inhibitors, also known as statins. This medication is used to treat high cholesterol and reduce the risk of cardiovascular disease [7]. This medication which is orally administered is recommended with lifestyle changes. As side effects, this medication can cause serious muscle damage, among other things, and also increases the risk of liver disease [8]. MVR is poorly soluble in water; according to the Biopharmaceutics Classification System (BCS) its solubility in water is 0.4 μg mL^−1^ [9]. It is highly permeable, and the rate of its oral absorption is often controlled by the dissolution in the gastrointestinal rate. When drugs are orally administered, their water solubility is one of the factors most affecting their efficacy [10], because a limited solubility of an active ingredient in water considerably reduces the dynamics of the drug absorption. Therefore, an enhancement in its dissolution in water leads to the improving of its bioavailability. Among the techniques used to improve the solubility of the lovastatin in water, solid dispersion has been shown to be an effective and practical method by different researchers in this field [11,12,13].

Poly(2-hydroxyethyl methacrylate) (PHEMA) swells but does not dissolve in water when its polymerization degree, $\bar{D}p$, is greater than 20 [14,15] and its swelling capacity is moderate (~40 wt%) [16]. With a $\bar{D}p$ lower than 20, this polymer is soluble at all temperatures. Those with $\bar{D}p$ localized between 20 and 45 exhibit cloud points. According to Armes et al. [15], the insolubility of PHEMA in water is mainly due to intra- and inter-chain H-bonding between polymeric hydroxy groups. The short chains may H-bond less efficiently, leaving a greater fraction of hydroxy groups to interact with water, or the H-bonding may simply not be sufficient to overcome the increased entropy of the short chains. As long as this polymer is soluble in other solvents such as dimethylformamide and dimethyl sulfoxide, this proves that the PHEMA is crosslinked physically (formation of inter-chain hydrogen bonds) and not chemically (formation of interchain covalent bonds).

PHEMA hydrogel was employed for the first time in the biological field by Wichterle and Lim [17]. Due to its safe tolerance, good biocompatibility, non-toxicity and non-antigenic properties, this hydrophilic material is widely studied in biomedical applications [18]. For example, this hydrogel was also developed as a carrier for water-soluble anticancer drugs, such as 5-fluorouracil [19], topical mitomycin-C [20], and cytarabine [21].

Poly(vinyl acetate) (PVAc) belongs to the family of shape memory polymers (SMP) which are smart materials that can be temporarily modeled and return to their original shape when an external stimulus is applied to them. PVAc is a hydrophobic and inexpensive polymer. It can be easily transformed into a hydrophilic biopolymer by hydrolysis in an acidic or basic medium by removing the molecules of acetic acid as a by-product giving poly(vinyl alcohol). This polymer, similarly to PHEMA, which is synthesized mainly by the free radical polymerization route, is biocompatible, biodegradable, non-toxic and non-carcinogenic [22,23,24]. However, its application as a shape memory polymer in the biomedical field is limited due to its weak mechanical properties such as the resistance modulus [22,25]. Due to its biological nature which respects the environment in which it is applied, it is used in various medical fields including cardiovascular devices, implants of artificial organs, cartilage skin and contact lenses. It is also used in wound dressings and various drug delivery applications [23,24].

In order to improve its mechanical properties and broaden its application in the medical field, PVAc is blended with others polymers having desirable properties [23,25,26,27] or employed as copolymers [28,29,30].

Several studies were performed to improve the dissolution properties and bioavailability of MVR, thereby increasing its drug efficacy. For example, Lin et al. [31] prepared nanoparticles involving poly(d, l-lactide-co-glycolide acid) (PLGA) and Mevacor using the double emulsion technique for its application in the dentistry field in the direct capping of pulp. It was found that these systems released MVR over a period of 44 days. In another study published by Tarafder et al. [32] on the release behavior of this same molecule from a coating of poly (ε-caprolactone) (PCL) on β-tricalcium phosphate (β-TCP), it was revealed that the hydrophilic-hydrophobic and hydrophobic-hydrophobic interactions that exist between MVR and PCL were the key factors controlling the diffusion dominated release kinetics of this medication from PCL coating. Recently, Madhavi et al. [33] studied the improvement of MVR absorption and its oral bioavailability. These researchers attempted to formulate floating drug delivery systems using Mevacor as a drug candidate using Methocel of different grades, e.g., K4 and K5. According to the results obtained, the F6 formulation containing 20 mg of MVR, xanthum gum was revealed as the optimized formulation and the result obtained was a release of more than 98.9% of this drug in 12 h.

To reduce the undesirable side effects of this very beneficial medication mainly used to treat high cholesterol and reduce the risk of cardiovascular disease, controlling the amount of MVR released, its uniformity, location, speed and duration of release must be well controlled.

In this work, to control the fraction of Mevacor released in a targeted medium over time, the reduction of the swelling rate of the PHEMA support and the improvement of its flexibility must be considered. To achieve this goal, a small amount of vinyl acetate unit (hydrophobic monomer) was incorporated into these PHEMA chains by copolymerization of HEMA with Vac comonomers. A series of Mevacor/poly(vinyl acetate-co-2-hydroxyethyl methacrylate) (MVR/VAC-HEMA) solid solutions with different MVR contents was prepared in one pot by the free radical photo-copolymerization reaction in the presence of this medication using camphorquinone as a photo-initiator under LED light. The structures of copolymer and solid solutions obtained were highlighted by FTIR and ^1^H NMR spectrometry and the distribution of MVR particles in the VAC-HEMA matrix was investigated by DSC, XRD and SEM techniques. The “in vitro” release kinetics were carried out in different pH media and the percentage of Mevacor released was evaluated by the UV-visible method. Different parameters influencing the release dynamics of this medication were widely studied such as the swellability of the copolymer material, the amount of the MVR loaded and the pH medium. Among others, the solubility enhancement of Mecavor in acidic and neutral pH media and the estimated distribution of the cumulative MVR released from MVR/VAC-HEMA hydrogel systems on the principal digestive organs timed according to Belzer et al.'s approach [34] were also investigated in this work.

## 2. Materials and Methods

### 2.1. Chemicals

Camphorquinone (CQ) (purity, 97%), vinyl acetate (VAc) (purity, 99%), and 2-hydroxyethyl methacrylate (HEMA) (purity, 99%) were supplied by Sigma Aldrich (KGaA, Germany). Mevacor (MVR) was provided from TCI America (Portland, OR, USA). Before usage, monomers were held under nitrogen gas after being purified by distillation at a reduced pressure. Photo-initiator (CQ) was used without purification. The human epithelial cell (HEC) line, GMSM-K prepared by Gilchrist et al. (2000) was offered by Dr Grenier of Laval Universitéy (Quebec, QC, Canada). Dulbecco’s Modified Eagle’s Medium (DMEM) was provided from Corning, Manassas, VA, USA. Fetal bovine serum (FBS) was purchased from Gibco (New York, NY, USA) and 1% of penicillin/streptomycin solution was supplied from Sigma Aldrich (St. Louis, MO, USA). A lactate dehydrogenase (LDH) detection kit was provided by BioVision (BioVision, Milpitas, CA, USA).

### 2.2. Preparation of PHEMA and PVAc Homopolymers

PHEMA and PVAc homopolymers were prepared by free radical polymerization of HEMA and Vac, respectively, under a lamp light led (6 × 5 watts) using CQ as photo-initiator. Initially, 5.35 g (0.041 mol) of HEMA, 3.530 g (0.041 mol) of VAc and 0.008 g of CQ were mixed in a beaker by stirring at an ambient temperature (~25 °C) until complete dissolution of the photo-initiator. The obtained solution was placed in a cylindrical mold made of Teflon and exposed to the LED lamp light during the polymerization reaction. The polymerization reaction was carried out under a moderated flux of nitrogen gas (3 mL·min^−1^) passing through the reactor fabricated as described by Alqahtani et al. [35,36]. The polymer was easily detached from the molder and dried in the open air for 12 h followed by the same duration in a vacuum oven at 40 °C. Both the resulting polymeric materials are a cylindrical sheet that is thin, transparent and flexible.

### 2.3. Preparation of MVR/VAC-HEMA Solid Solution

**Scheme 2:**
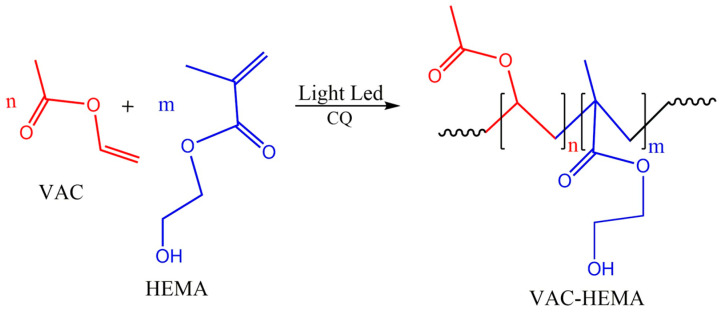
Free radical copolymerization reaction of HEMA with VAC.

MVR/VAC-HEMA solid solution was prepared in one pot by free radical copolymerization of Vac with HEMA in presence of MVR as filler and CQ as photo-initiator. First, 0.041 mol (5.35 g) of HEMA and 0.007 mol (0.62 g) of VAc were mixed in a beaker by stirring at an ambient temperature (~25 °C), then 0.008 g of CQ was added to the reaction mixture. The copolymerization reaction was carried out as shown in Scheme 2 in the same conditions as described in the Section 2.2. As in the previous case, the copolymer was easily detached from the molder and dried in the open air for 12 h followed by a same duration in a vacuum oven at 40 °C. The resulting polymeric material is a cylindrical sheet that is thin, transparent and flexible. A series of MVR/VAC-HEMA containing 0.5 wt% (0.03 g), 1.0 wt% (0.06 g), 1.5 wt% (0.09 g) and 2.0 wt% (0.12 g) MVR contents was prepared by this same procedure.

### 2.4. Characterization

#### 2.4.1. FTIR Analysis

A Perkin Elmer Spectrum GX FTIR spectrometer (Waltham, MA, USA) operating in attenuated total reflection mode, in a wave number range of 4000 to 650 cm^−1^. Thirty-two (32) cycles of scanning, and a resolution of 2 cm^−1^ was used to record the Fourier transform infrared (FTIR) spectra of pure MVR, PVAc, PHEMA homopolymers, and VAC-HEMA copolymer. The samples were analyzed in the form of thin transparent films except that of MVR which was analyzed as powder diluted in dehydrated KBr.

#### 2.4.2. HNMR Analysis

A JEOL FX 90 Q NMR spectrometer, (Tokyo, Japan) at 500 MHz was used to characterize the ^1^HNMR copolymer and their corresponding homopolymers were dissolved in DMSO-d_6_.

#### 2.4.3. SEC Analysis

The SEC chromatogram of VAC-HEMA was performed on a Varian apparatus equipped with JASCO type 880-PU HPLC pump, refractive index, UV detectors and TSK gel columns calibrated with polystyrene standards. Dimethylformamide (DMF) was used as an eluent at 30 °C.

#### 2.4.4. DSC Analysis

The DSC thermograms of MVR, virgin VAC-HEMA and MVR/VAC-HEMA solid solutions containing different MVR contents were plotted on a DSC device (Shimadsu DSC 60, Tokyo, Japan) previously calibrated with indium. Between 10 and 12 mg of samples were packaged in aluminum DSC capsules before being placed in the DSC cell. Samples were scanned by heating from −25 to 250 °C with a heating rate of 10 °C∙min^−1^. The glass transition temperatures (Tgs) of homopolymers, copolymer and copolymers containing medication with different contents were taken from the inflection point of the thermal curves, which characterizes the sliding of about 50% of the polymer chains. The melting temperature of MVR powder was determined from the top of the endothermic peak corresponding to its enthalpy of fusion.

#### 2.4.5. XR-Diffraction Analysis

An advanced BRUKER D8 diffractometer (Germany) was used to perform the samples X-ray spectrographs of pure and mixed materials. Patterns were run with Cu Kα radiation at 40 mA and 40 kV with a 2θ scan rate of 2° min^−1^.

#### 2.4.6. SEM Analysis

Before and after the release procedure, the micrographs of MVR powder, virgin VAC-HEMA, and MVR/VAC-HEMA dried films of various MVR contents coated with gold grid were investigated by scanning electron microscope (SEM) using a Hitachi S4700 Field emission (Tokyo, Japan).

#### 2.4.7. Cell Toxicity and Cell Adhesion

According to Semlali et al. [37], the LDH assay was used to assess the cellular cytotoxicity of all specimens. On 24-well plates for 24 h, 2 × 10^5^ cells containing a specimen were seeded. Fifty (50) µL of duplicate LDH mix solution was added to an equal volume of each supernatant. These solutions were placed in a 96-well plate and incubated for 20 min at room temperature in the dark until the yellow color appeared. The reading was taken at 490 nm with an iMark Microplate Absorbance Spectrophotometer (Bio-Rad, Mississauga, ON, Canada). A percentage was used to represent the cell toxicity. Next, 2 × 10^5^ cells treated with 1% of Triton X-100 were used as a positive control for LDH and which corresponds to 100% cytotoxicity. This experiment was replicated three times for each specimen.

According to our previous works, the MTT test was used to measure cell adhesion for each specimen placed on 24-well plates [38,39]. Briefly, 2 × 10^5^ GMSMK cells/specimen were seeded in 24-well plate, cultured for adhesion overnight. Each cell culture was then given a 1/10 volume addition of a 5 mg∙mL^−1^ MTT solution for 3 h of incubation at 37 °C in the dark before using 300 µL of isopropanol 0.05N of HCL solution to lyse the cells. Next, 2 × 100 µL of lysed solution was poured into a 96-well microplate to assess absorbance at 550 nm by an iMark reader (Bio-Rad, Mississauga, ON, Canada). The percentage cell viability was used to assess the percentage of viable, proliferating cells. In addition, 0% adhesion corresponds to Absorbance (A) at 550 nm for a cell-free specimen.

#### 2.4.8. Swellability

At 37 °C, distilled water was used to test the copolymer hydrogel’s swelling degree until saturation (swelling equilibrium). A square film sample of copolymer measuring 3 cm × 3 cm × 3 mm was completely immersed into a beaker that had been filled with 100 mL of distillate water. The entire system was kept at a temperature of 37 °C with moderate agitation. After being withdrawn from the beaker at regular intervals, the specimen was gently wiped of the droplets that had been left on the film specimen’s two surfaces with absorbent paper before being weighed on a precision balance. The identical conditions were used to conduct this experiment three times, and the swelling degree, *SD* (*wt*%), was calculated from the mean arithmetic values obtained using Equation (1)

(1) $$SD\left(wt\%\right)=\frac{m_{t}-m_{o}}{m_{o}}\times 100,\;$$

where $m_{o}\;$ and $m_{t}\;$ are the masses of the film specimen weighted before and at *t* time of the swelling process, respectively.

#### 2.4.9. In Vitro Release Dynamic

MVR/VAC-HEMA film samples of different MVR contents were immersed separately in 20 mL of bi-distilled water maintained at pH levels of 1, 3, 5, and 7 at body temperature (37 °C) for 72 h with a stirring rate of 100 rpm. To monitor the amount of MVR released, aliquots of 0.5 mL are taken at time intervals and immediately returned to the media after analysis. This operation allows for a constant volume of media during the release process. The concentration of MVR released during a given period time was determined by UV-visible analysis using a double beam U-2910 Hitachi spectrophotometer(New York, NY, USA).

The cumulative mass of MVR released was obtained from the calibration curve indicating the variation in medication concentration versus absorbance. A buffer solution does not need to be added to the water in this situation since the small amount of MVR produced (pKa = 13.49) [36] during the release process has practically no impact on the stability of the pH medium.

## 3. Results and Discussion

### 3.1. Characterization

#### 3.1.1. FTIR Analysis

**Figure 1:**
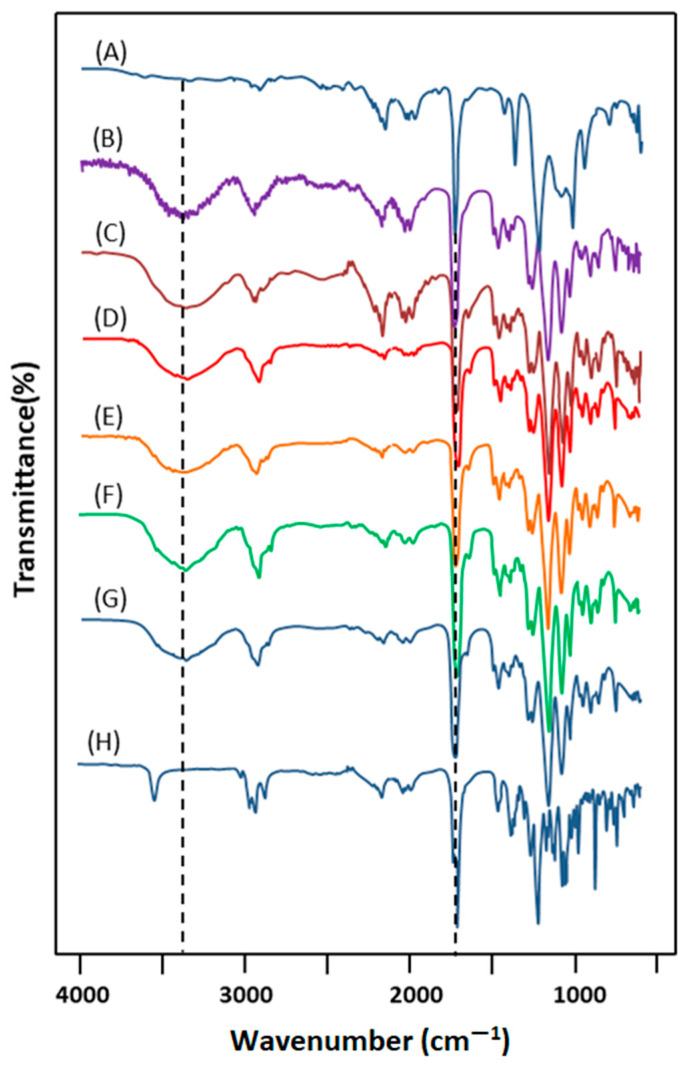
FTIR spectra of PVAc (A), PHEMA (B) and VAC-HEMA containing 0 (C), 0.5 (D), 1.0 (E), 1.5 (F), 2.0 (G) wt% MVR and (H) pure MVR. (---) Reference bands compared to those of pure PHEMA.

The FTIR spectra of pure MVR, virgin VAC-HEMA and MVR/VAC-HEMA solid solution with different MVR contents are gathered in Figure 1. The comparison of the spectrum of VAC-HEMA with those of PHEMA and PVAc reveals the presence of the combination of the signals attributed to these two homopolymers. The absorption band of the signals attributed to the carbonyl groups of the HEMA and VAc units is observed at 1704.70 cm^−1^ and that of the hydroxyl group at 3365.04 cm^−1^. Given the small amount of MVR loaded into the MVR/VAC-HEMA system, the spectra of the solid solution containing 0.5 and 1.0 wt% of this medication do not show additional signals characterizing the structure of this drug, such as that of the signal at 801 cm^−1^ which is assigned to the δ(CH) rocking mode [40]. The comparison of these spectra also reveals shifts in the carbonyl groups from 1704.70 to 1716.08 cm^−1^ and another shift in the hydroxyl groups of the copolymer from 3365 to 3358.27 cm^−1^, thus revealing the presence of hydrogen bonds between the hydroxyl and the carbonyl groups of the HEMA unit and those of MVR medication. This confirms the uniform dispersion of this medication in its molecule level in the copolymer matrix.

#### 3.1.2. NMR Analysis

**Figure 2:**
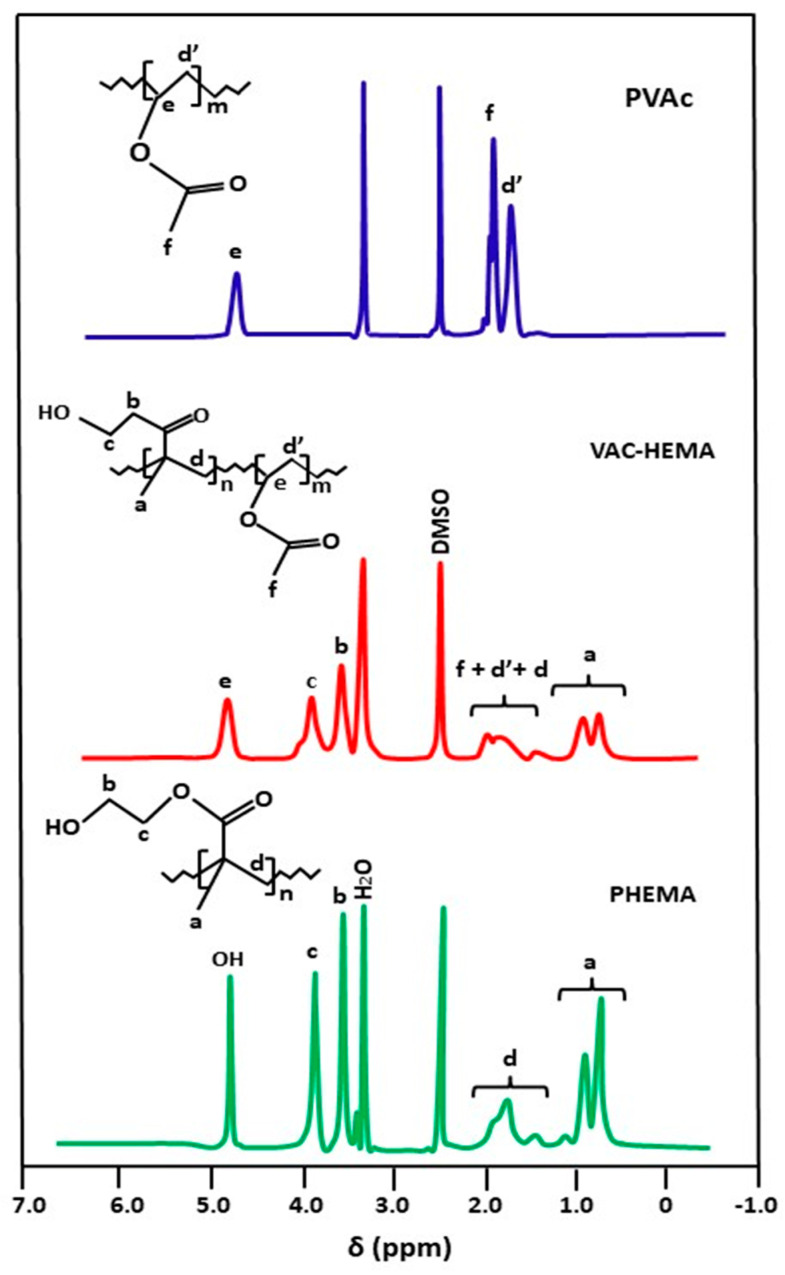
^1^HNMR spectra of PHEMA, PVAc and VAC-HEMA copolymer.

The structure of the VAC-HEMA was demonstrated by ^1^H NMR analysis in the light of the comparison of its spectrum with those of their corresponding PHEMA and PVAc homopolymers as shown in Figure 2. Indeed, the spectrum of VAC-HEMA overlap the signals of the two different homopolymers, thus indicating the presence of the two monomers involved in the copolymerization reaction. The multiplets that appear between 0.35 and 1.0 ppm are assigned to the methyl group of the HEMA sequence isomers in the copolymer chain, and those between 1.3 and 2.1 ppm are attributed to the ethylenyl groups of the isomers of the two different units involved in the copolymer [41]. The percentage of the VAc and HEMA monomeric units in the VAC-HEMA copolymer was determined on the basis of the areas of the signals attributed to the three protons of the methylene group (a) and those of the signals of the ethynyl group (*d* + *d′*) common to the two different monomeric units using Equation (2),

(2) $$HEMA\left(mol\%\right)=\frac{2\delta_{CH_{3}\left(a\right)}}{3\delta_{CH_{2}\left(d+d^{\prime }\right)}}\times 100$$

where $\delta_{CH_{3}\left(a\right)}$ and $\delta_{CH_{2}\left(d+d^{\prime }\right)}$ are the surface areas of the three protons of –*CH*_3_ (a) and the two protons of –*CH*_2_-(*d* + *d*’) common to the two monomeric units, respectively. The determination of the composition in HEMA units in the VAC-HEMA copolymer indicates 84.22% by mole taken as the arithmetic mean of three spectra for this same copolymer. This composition is desirable in order to maintain the affinity of the MVR with the resulting material (miscibility) while limiting its swelling to a rate allowing it to be used as an adequate carrier in the ACR/VAC-HEMA drug carrier system.

**Figure 3:**
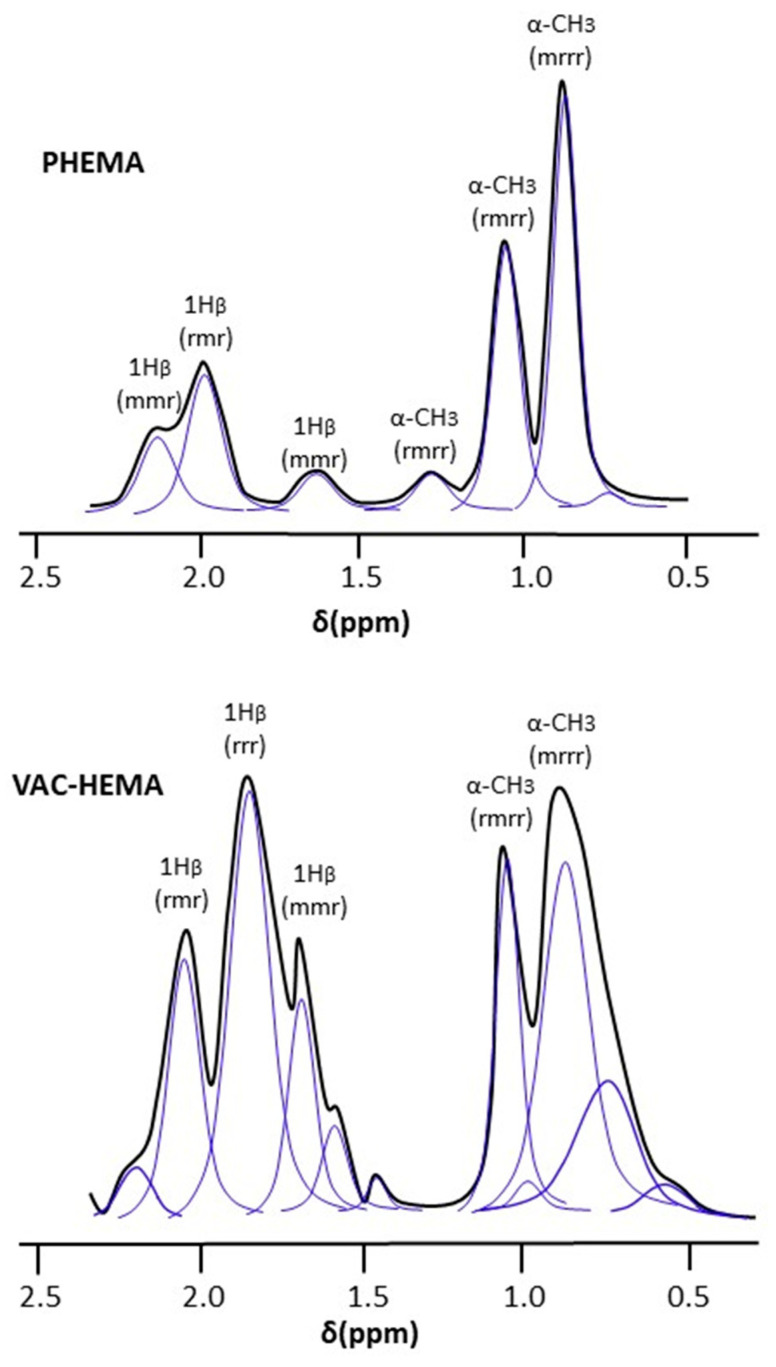
Decomposition in Lorentzian curves of the ^1^HNMR signals of PHEMA and VAC-HEMA between 0.5 and 2.3 ppm.

The deconvolution of these signals between 0.5 and 2.3 ppm in Laurentzien curves, as shown in Figure 3, permitted to determine the average tacticity (%) of the triad, tetrahedral and pentahedral sequences distributed in the VAC-HEMA chains. The results obtained revealed that this copolymer has a mainly heterotactic microstructure with atactic pentaeds *mrrr* (31.26%), *rmrr* (15.72%) and *mmmr* (10.14%), and the rest are mainly syndiotactic tetraheds *rrr* (21.42%) and atactics *rmr* + *mmr* (11.62%).

These values were taken from the calculations of triplicate spectra for each sample, and the results obtained revealed that the microstructure of VAC-HEMA copolymer obtained practically reflects that of the PHEMA homopolymer which is mainly heterotactic as shown in this figure. These results are not surprising, because it is well known that in general, the polymerization of vinyl or acrylic monomers by the free radical route leads to mainly heterotactic microstructures due to the uncontrollable free radicals resulted during the reaction.

#### 3.1.3. XR-Diffraction

**Figure 4:**
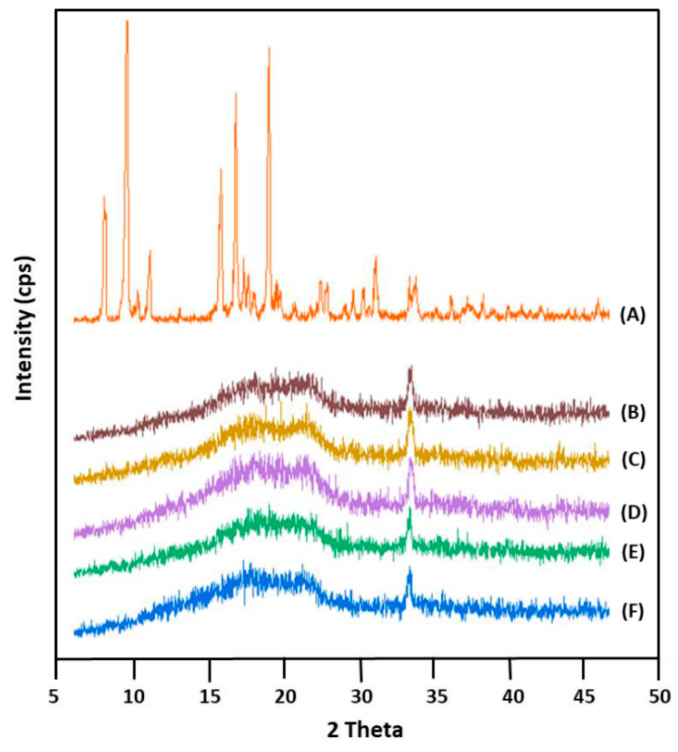
XR-Diffraction patterns of: (A) MVR powder; (B) VAC-HEMA; (C) MVR/VAC-HEMA0.5; (D) MVR/VAC-HEMA1.0; (E) MVR/VAC-HEMA1.5 and (F) MVR/VAC-HEMA2.0.

The XR-Diffraction spectra of MVR powder, virgin VAC-HEMA and MVR/VAC-HEMA solid solutions are grouped for comparison in Figure 4. The spectrograph of the virgin VAC-HEMA shows an amorphous structure by the absence of sharp signals characterizing a crystalline structure. The spectrum of MVR powder shows a crystalline structure through the appearance of sharp signals at 7.90, 9.31, 11.35, 15.00, 16.80, 19.21 and 26.50° 2 theta, which agree with the literature [12]. The disappearance of these signals in the spectra of MVR/VAC-HEMA systems indicates that the MVR are homogeneously dispersed in the VAC-HEMA matrix in its molecular level. Noting that, the presence of aggregated particles in an amorphous polymer is evidenced by the appearance of signals specific to the crystalline structure of the filler in the spectrum of the MVR/VAC-HEMA solid solution or by their shift in the event of modification of the geometry of the resulted crystals.

#### 3.1.4. SEC Analysis

**Figure 5:**
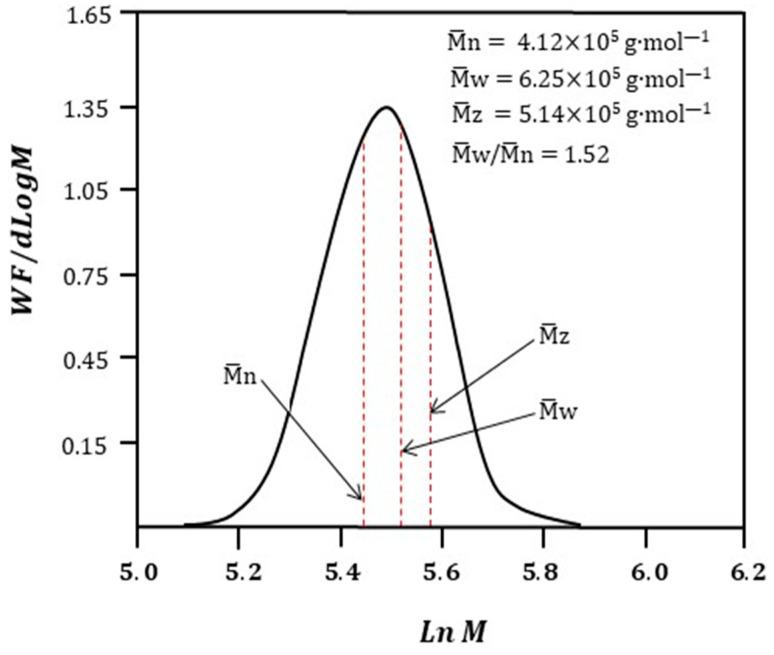
Chromatogram SEC of VAC-HEMA obtained at 30 °C in DMF used as eluent at 30 °C.

The SEC chromatogram of VAC-HEMA in Figure 5 indicates a relatively high molecular weight ($\bar{M}n=$4.12 × 10^5^ g∙mol^−1^ and $\;\bar{M}w$ = 6.25 × 10^5^ g∙mol^−1^) characterized by a relatively narrow molecular distribution, in which the polydispersity index (PDI) was 1.52. This indicates that there was an important reduction in the transfer reactions during the copolymerization reaction. Note that a large PDI indicates the presence of a small fraction of low macromolecular weight in the polymers. According to different authors [15,16], PHEMA chains having degrees of polymerization lower than 20 are soluble in water at all temperatures. This is not desirable as a carrier for use in the drug release fields. The incorporation of 15.78 mol% of VAC hydrophobic units in the PHEMA chains considerably reduce the water solubility of this copolymer.

#### 3.1.5. DSC Analysis

**Figure 6:**
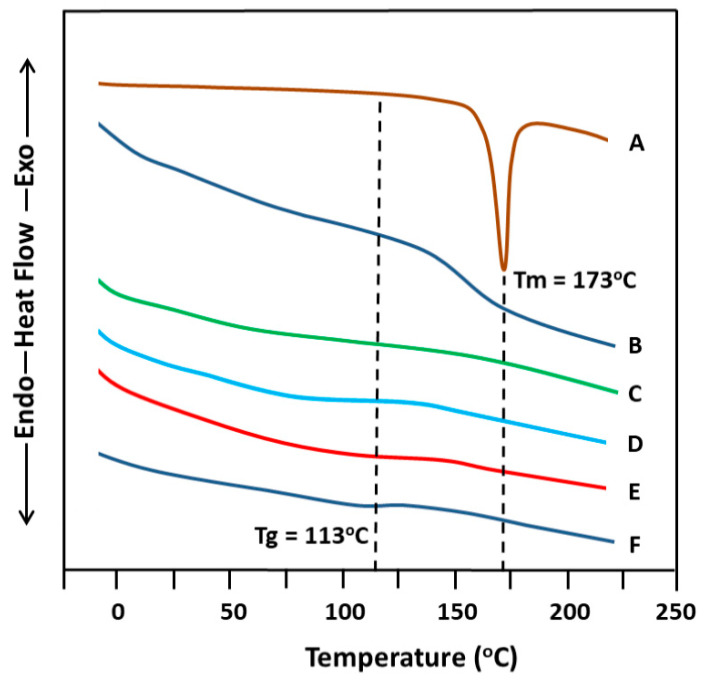
DSC thermograms of MVR (A), virgin VAC-HEMA (B) and MVR/VAC-HEMA containing 0.5 wt% (C), 1.0 wt% (D), 1.5 wt% (E) and 2.0 wt% (F) MVR.

The DSC curves of the MVR/VAC-HEMA solid solutions and their pure components are grouped together in Figure 6. As can be observed from the thermogram of pure MVR, this medication shows a melting temperature at 173 °C, which agrees with the literature (173.4 °C) [11] and the profile of the thermal curve of the virgin VAC-HEMA shows a Tg of 113 °C for this copolymer. On the other hand, when 0.5 wt% of MVR was incorporated in the VAC-HEMA matrix, a drop in the Tg of 25 °C was observed which passed from 113 to 88 °C. As the content of MVR incorporated into the copolymer becomes greater, a significant increase in the Tg of the copolymer is noted reaching a maximum of 145 °C when 2.0 wt% of this drug was loaded. Regarding the MVR/VAC-HEMA systems, the total disappearance of the endothermic peak characterizing the fusion of MVR in their thermograms indicates that this drug is indeed uniformly dispersed in the copolymer matrix in its molecular level. This confirms the formation of a solid solution involving the MVR as solute and the VAC-HEMA as solvent.

#### 3.1.6. SEM Analysis

**Figure 7:**
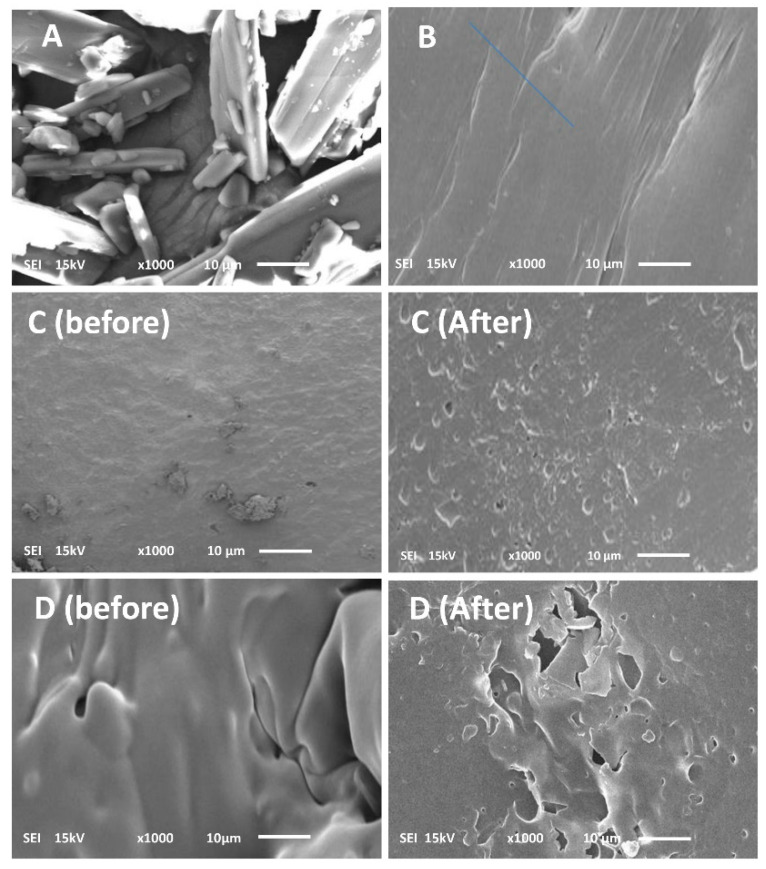
SEM micrographs of MVR (**A**), VAC-HEMA (**B**) and MVR/VAC-HEMA drug carrier systems containing: 0.5 wt% (**C**) and 2.0 wt% (**D**) MVR before and after the release process.

The surface morphologies of the MVR/VAC-HEMA drug carrier systems and their pure components were examined by the scanning electron microscopy and the images of pure MVR, virgin VAC-HEMA and their mixtures containing 0.5 and 2.0 wt% medication are shown as examples in Figure 7. The image of the pure MVR (A) shows smooth rectangular rod-shaped particles on the corners of different dimensions. A similar photo of this medication was also obtained by Guan et al. [42]. The micrograph of the virgin VAC-HEMA (B) shows a smooth, slightly rugged surface devoid of any particles deposited on the surface, resembling a snowy mountain descent. Before the release process, the image of the MVR/VAC-HEMA0.5 system (C) shows a slightly undulating surface resembling muddy ground. As can also be seen in this photo, this surface is devoid of any particles deposited or embedded on the copolymer material. This indicates that the Mevacor particles are well dispersed homogeneously in their molecular level in the VAC-HEMA matrix. This confirms the results obtained by DSC and XR-D analysis. This same film sample, after the release process, shows a surface containing a high density of well-dispersed circular and oval-shaped pores with an average diameter varying between 0.3 and 16 µm, testifying to a massive release of MVR medication. On the other hand, before the release process, the image of MVR/VAC-HEMA2.0 drug carrier system, which contains the maximum amount of MVR (D), shows a smooth surface containing plaice formed during the vacuum drying of the sample. After release, this same sample, as in the case of the MVR/VAC-HEMA0.5 specimen, shows a high density of pores, but sometimes of a much larger size testifying to the release of a significant amount of drug.

#### 3.1.7. Swellability

**Figure 8:**
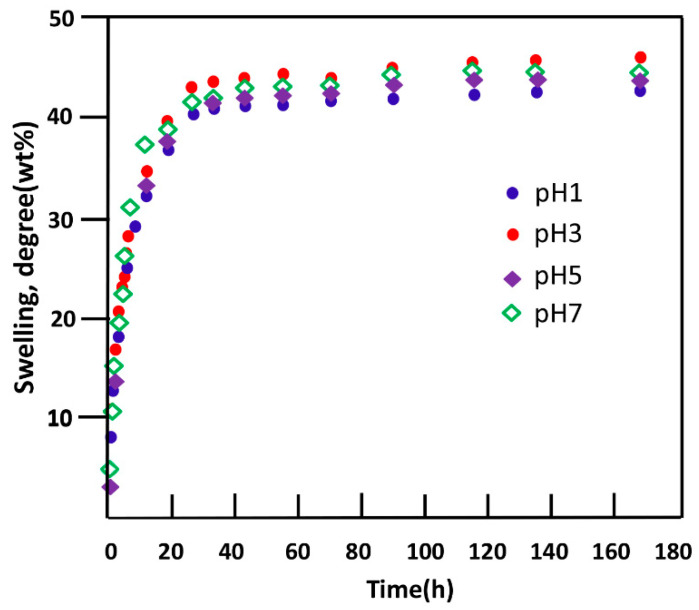
Swelling behavior of virgin VAC-HEMA material in water at 37 °C.

To study the dynamics of a drug released from a drug carrier system in an aqueous media, knowledge of the swellability of a material used as a carrier is a key factor that must be taken into account. According to the literature, at body temperature (37 °C), this parameter is influenced by different factors such as the nature of the used carrier, the crosslinking degree of the polymer [43,44] and the pH medium in which the drug will be released [45,46]. In this work, the variation in the swelling degree of VAC-HEMA copolymer versus time, carried out at 37 °C during one week in different pH media, is plotted in Figure 8. As can be observed from the profiles of these curves obtained after triplicate experiments, usual exponential functions are obtained and the values of the swelling capacity of the specimen in pH media 1, 3, 5 and 7 varied between 42.2 ± 0.3 and 45.8 ± 0.5 wt%, which reveal no significant change in the swelling capacity of VAC-HEMA material in this pH range. This result was predicted, because the nature of the copolymer in question does not a seat of acid-base interaction with the pH medium. Based on this principle, the release dynamics of the MVR will depend only on a synergy involving the properties of VAC-HEMA copolymer and those of the medication loaded and not on the pH medium.

#### 3.1.8. Water Diffusion

According to Comyn [47], the diffusion of small molecules such as water through a polymer material for a short period of diffusion and when the *wt*/$w_{\infty }$ is less than 0.5 is expressed by the following relationship:

(3) $$\frac{w_{t}}{w_{\infty }}=2{\left(\frac{D}{\pi l^{2}}\times t\right)}^{0.5},\;$$

where $w_{t}\;$ and $w_{\infty }$ are the weight of the sorbed molecules during a *t* time and the total weight absorbed, respectively. *l* and *D* are the polymer film thickness and the diffusion coefficient of the molecules sorbed, respectively. The linearization of Equation (4) leads to Equation (5)

(4) $$Ln\left(\frac{w_{t}}{w_{\infty }}\right)=lnk+nLnt,\;\mathrm{with}\;k=\frac{2}{l}{\left(\frac{D}{\pi }\right)}^{0.5},$$

**Figure 9:**
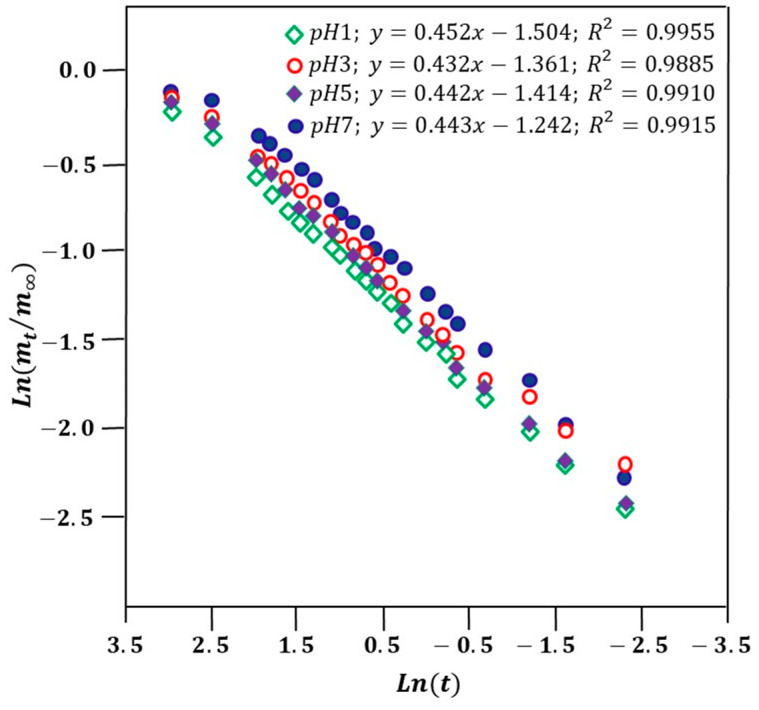
Variation in $Ln\left(m_{t}/m_{\infty }\right)$ of virgin VAC-HEMA hydrogel versus *Ln*(*t*) at 37 °C and in different pH media.

The slope of the linear portion of the curve indicating the change in the $\;Ln\left(\;w_{t}/w_{\infty }\right)$ as function of the square root of time (Figure 9) give the *D* value of water molecules through the VAC-HEMA hydrogel. Indeed, the profile of the curves obtained reveals straight line for this hydrogel in all pH media investigated. This reveals that the diffusion of water molecules through the VAC-HEMA matrix obeys a Fickian model. The basic equation allowing to obtain the mass of molecules absorbed by a polymeric material is given by the following equation [48],

(5) $$\frac{w_{t}}{w_{\infty }}=kt^{n},\;$$

where *n* and *k* are the type of the diffusion mechanism and a constant relating the swelling rate, which depends on the film thickness and the diffusion coefficient, respectively. In this work, the *n* and *k* values are deducted from the slope and the intercept of these linear curves and the results obtained are gathered in Table 1.

In general, the values of *n* are close to those of the exponent in Equation (6), thus characterizing the order 0.5 of the diffusion of water molecules in the VAC-HEMA carrier. On the other hand, the values of *k* and *D* slowly increase with the pH of the medium. This indicates that, during the swelling process, the water diffusion rate increased with the pH of the medium and reached about 1.5 times that in the neutral medium. This seems to indicate that in a neutral medium the hydrogen bonds between the molecules of water and those of the copolymer are more favorable. This can be interpreted as a small reduction in the hydrophilicity of the copolymer due to a reduction in the density of the inter- and intra-chain hydroxyl bonds of the copolymer in acidic media. This is probably due to intra- and inter-chain etherification reactions involving two adjacent hydroxyl groups of the HEMA units that result in the elimination of water molecules by forming hydrophobic ether bonds. Consequently, this leads to a reduction in the diffusion rate of water molecules inside the copolymer matrix.

#### 3.1.9. MVR Solubility Enhancement

**Scheme 3:**
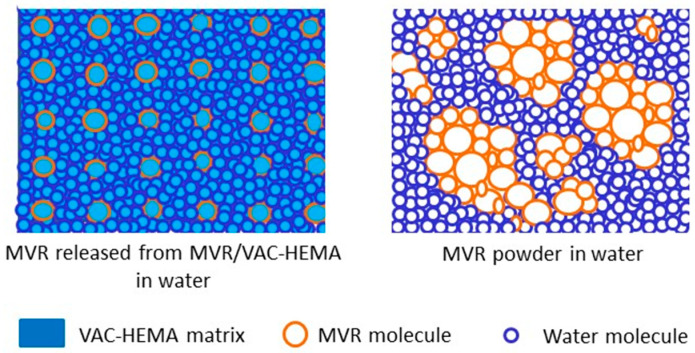
Solubility enhancement of MVR.

The maximum solubility of MVR in media pH 1 and 7 was deduced from the maximum amount of this medication released from the MVR/VAC-HEMA drug carrier systems over time using the shake flask method [49]. The solubility of MVR powder in water at pH 1 and 7 was measured at saturation characterized by the beginning of the appearance of turbidity in which the reflectance in the UV-visible range increased sharply indicating a supersaturation of the resulted solution. The supersaturated solution is centrifuged at ambient temperature (~25 °C) and the liquid supernatant is filtered. To avoid crystallization of MVR in the medium, 0.5 mL of the supernatant is taken and then diluted with the same medium. The measurements of the absorbance and reflectance were performed at 37 °C and the solubility at equilibrium of MVR in neutral pH and pH medium 1 were taken from the arithmetic average of three experiments. The maximum solubility deducted from the maximum release of MVR from the MVR/VAC-HEMA drug carrier systems in these same media are gathered with that dissolved directly as powder in Table 2. As can be seen from these data, the maximum solubility of MVR increased significantly when dispersed at its molecular level in the VAC-HEMA matrix (Scheme 3). Indeed, the solubility of this same medication obtained by this method increased more than 22 and 37 times that dissolved as powder in media pH 1 and pH 7, respectively. This is due to a significant increase in the contact surface between the dispersed MVR molecules and those of the aqueous medium inside the carrier matrix. According to the results obtained on the physico-chemical and thermal characterization of MVR/VAC-HEMA systems by XRD (Section 3.1.3) and DSC (Section 3.1.4), it was revealed that the MVR was effectively homogeneously distributed in the carrier matrix in its molecular state, this implies that the solvation of this drug was in its maximum state.

#### 3.1.10. Cytotoxicity

**Figure 10:**
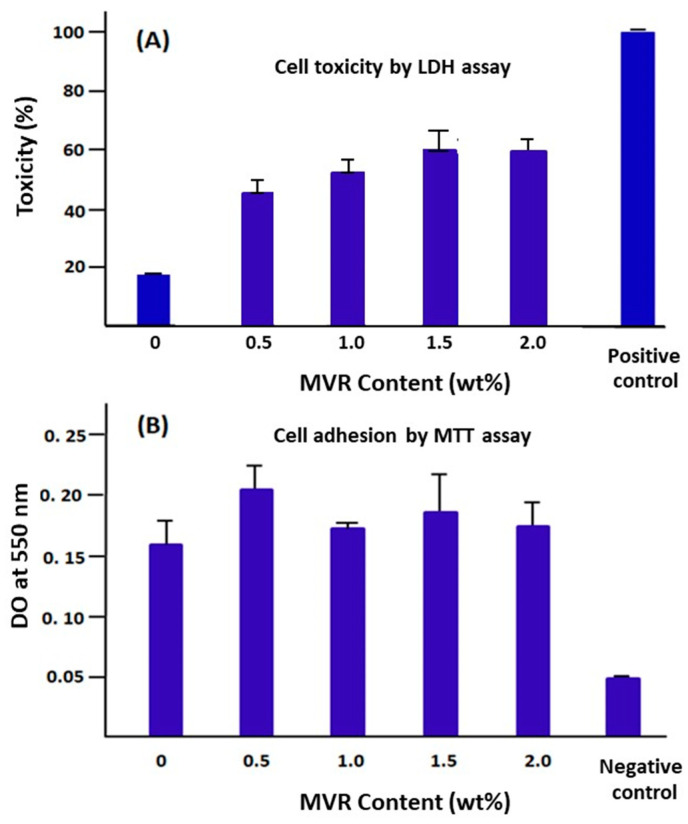
(**A**) The effect of MVR/VAC-HEMA materials on the HEC toxicity examined by the LDH assay and (**B**) HEC adhesion on the virgin VAC-HEMA and MVR/VAC-HEMA specimens. The positive control used in this experiment is the collagen membrane (collapse) giving 100% adhesion.

The cell toxicity of VAC-HEMA and MVR/VAC-HEMA materials was measured at 490 nm from triplicated tests and the percentage toxicity taken from their average values is visualized in a histogram in Figure 10A. As can be observed from these results, the normal HEC appear to be unaffected by the virgin specimen (VAC-HEMA) and no significant effect on their viability is observed when between 0.25 and 2.00 wt% was loaded in this material. Although the cytotoxicity of this material was not significant, increasing the MVR content in the MVR/VAC-HEMA system from 0.25 to 2.00 wt% leads to increase the cell cytotoxicity from 19.2 to 58.3%. This appears to be due to the effect of this drug as an antiviral agent on healthy cells.

#### 3.1.11. Cell Adhesion

The cell adhesion testing on virgin VAC-HEMA and MVR/VAC-HEMA drug carrier systems was performed by the standard MTT assay and the results obtained were taken from triplicated tests and the average percentages of cell adhesion for each sample are presented in the histogram of Figure 10B. These data, compared to the negative control, indicate a significant cell adhesion rate for all prepared samples. These data also reveal that the absorbance of the virgin VAC-HEMA increased by more than three fold that without cells, however no significant difference is noted when this hydrogel is loaded with 0.25 to 2.00 wt% of the MVR content.

### 3.2. In Vitro Release Dynamic

For non-porous (dense) and asymmetric drug-carrier systems, the mechanism of a drug released in aqueous medium involves three principal phenomena: (i) the penetration of the release medium into the drug-support system takes place under the action of the osmosis phenomenon (the water passes from the least concentrated medium to the most concentrated medium), and its rate of diffusion depends mainly on the affinity between the release medium and the drug-carrier system, (ii) the dissolution of the drug inside the drug-carrier system and this depends mainly on its solubility in this medium, (iii) the release of the drug which is performed in solution under the action of a thermodynamic imbalance caused by the large difference between the free enthalpies of the mixture inside and outside the drug-carrier system due to the large difference between the drug concentration inside and outside the system.

**Figure 11:**
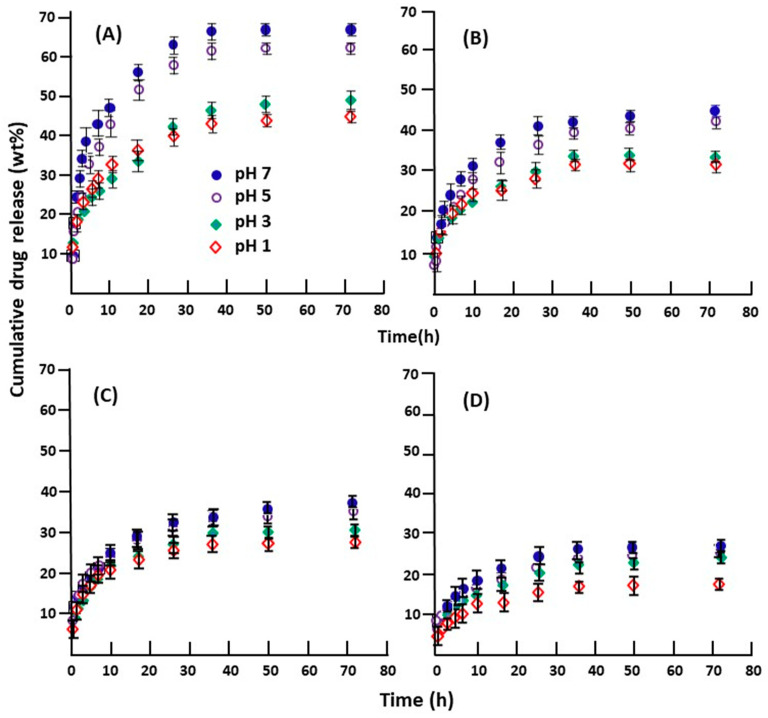
Change in the cumulative MVR released from the MVR/VAC-HEMA drug carrier systems containing 0.5 (**A**), 1.0 (**B**), 1.5 (**C**) and 2.0 (**D**) wt% of MVR versus time.

In this present work, the release of MVR from the MVR/VAC-HEMA drug carrier systems was carried out at 37 °C during 72 h in media pH 1, 3, 5 and 7, separately, and the results obtained are plotted in Figure 11. For all samples, the release dynamics of this drug follows a logarithmic pattern with a variable slope depending on the MVR content in the drug carrier system. These data reveal, for any pH medium, that the maximum cumulative of MVR released is reached with the MVR/VAC-HEMA0.5 specimen containing the lower percentage of MVR initially loaded (0.5 wt%) in the copolymer matrix. In terms of the amount released, the maximum drug release is obtained with the system that contains the most MVR loaded in the VAC-HEMA carrier (2.0 wt%). The decrease in the percentage of the drug released when the initial amount of MVR loaded in the VAC-HEMA matrix seem to be due to the limit solubility of MVR in water inside the copolymer matrix, because a small amount of medication is much easier to dissolve than a larger amount. As can be seen from these results, for all MVR/VAC-HEMA compositions, the release percentage of this medication decreased with the pH of the medium. The explanation of this phenomenon will be amply presented in Section 3.3.

#### 3.2.1. Diffusion Behavior of MVR

In order to understand the release dynamics of MVR over time, the diffusion behavior of this medication through MVR/VAC-HEMA drug carrier systems was investigated. The MVR released from these materials in different pH media does not exceed 60 wt% of this medication initially loaded in these drug carrier systems. Therefore, the Fickian model is applicable to describe the diffusion mechanism of MVR through the VAC-HEMA material. According to Masaro et al. [48], the relationship between the amount of drug released as a function of time resulting from the Fick model is given by Equation (6)

(6) $$\frac{m_{t}}{m_{o}}=k^{\prime }\sqrt{t}$$

where $m_{t}/m_{0}$ and $k^{\prime }$ are the weight ratio of MVR released at *t* time of the release process and a constant related to the drug carrier system, respectively.

**Figure 12:**
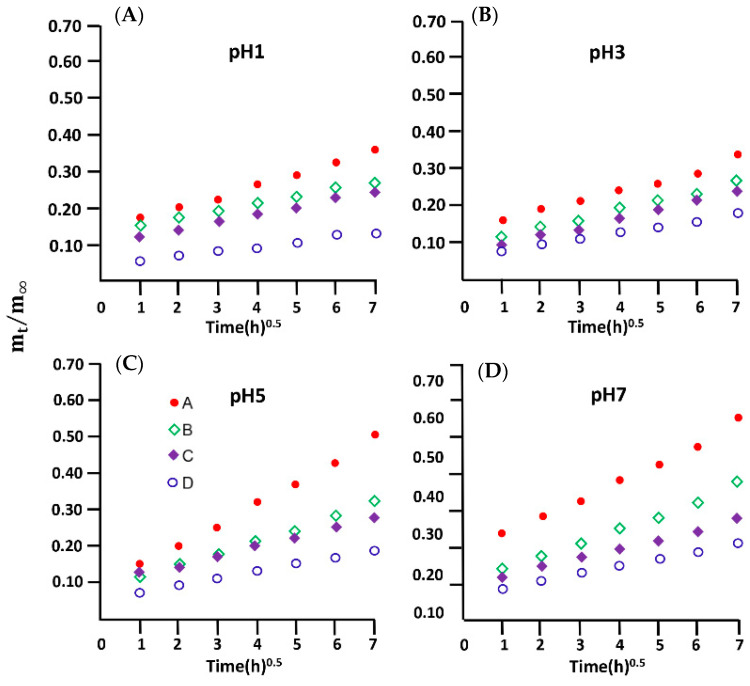
Variation in the drug fraction released from MVR/VAC-HEMA systems containing 0.5 (**A**), 1.0 (**B**), 1.5 (**C**) and 2.0 (**D**) wt% of MVR in different pH media versus the square root of time.

If the drug molecules diffuse through the MVR/VAC-HEMA system according to the Fick model, the graph indicating the variation in the drug fraction released $m_{t}/m_{0}$ as a function of the square root of time would give a straight line with a slope corresponding to the value of the *k′* constant. Once the *k′* for a drug carrier system is determined, the *D*’ value is easily calculated using Equation (7):

(7) $$k^{\prime }=4\sqrt{\frac{D^{\prime }}{\pi l^{2}}}\;,$$

where *l* is the thickness of the MVR/VAC-HEMA film sample. As shown in Figure 12, the variation in the weight fraction of the MVR $(m_{t}/m_{0})\;$ released as function of the square root of time describes a straight line and the *k’* and *D’* values, deducted for all MVR/VAC-HEMA drug-carrier systems, are gathered for comparison in Table 3. As can be seen from these data, the *D’* value of the MVR molecules through the MVR/VAC-HEMA drug carrier system increased slowly with the MVR content.

#### 3.2.2. Effect of the Initial Amount of MVR

**Figure 13:**
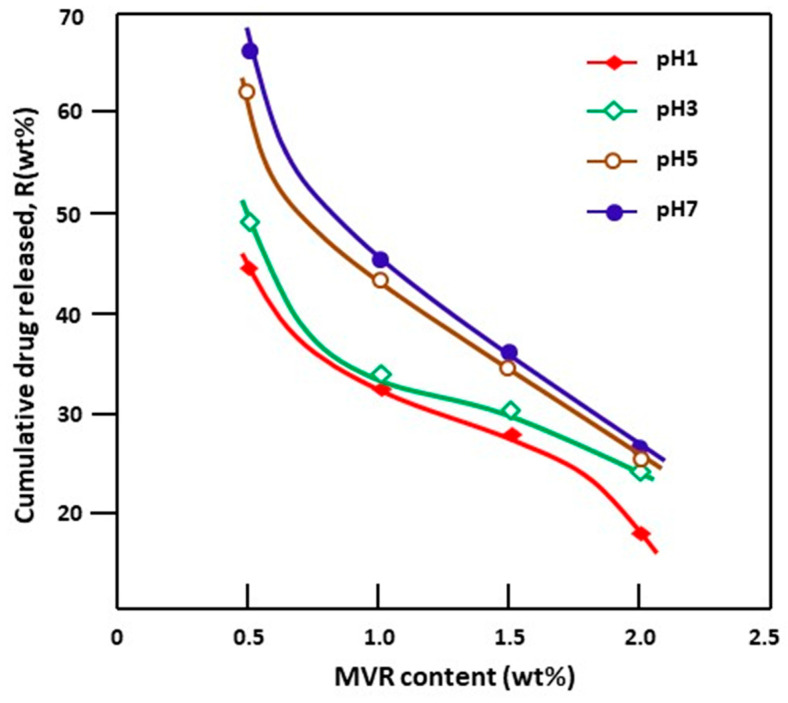
Cumulative MVR released form the MVR/VAC-HEMA drug carrier system versus the MVR content loaded in the VAC-HEMA matrix obtained over 3 days of the release process.

The effect of the MVR amount initially loaded in the VAC-HEMA carrier was carried out over 3 days in different pH media and the results obtained are presented in Figure 13. The profile of the curves obtained shows a significant decrease in the MVK released in any pH medium when this medication initially loaded in the VAC-HEMA increased. This is undoubtedly due to the limited solubility of MVR in water inside the VAC-HEMA matrix, which was found in this investigation to equal 0.131 and 0.141 mg∙mL^−1^ in pH media 1 and 7, respectively. Indeed, as demonstrated in Section 3.1.6, the swelling capacity of the VAC-HEMA carrier remains unchanged regardless of the pH medium. Therefore, the concentration of the dissolved drug inside the VAC-HEMA matrix also remains unchanged regardless of the amount of MVK initially loaded into this material. Therefore, an excess of this medication leads to decrease the percentage released. This can also be interpreted by the diffusion coefficient of this drug in these different media inside the copolymer matrix (Table 3). Indeed, this parameter was faster in the case of drug carrier systems containing 0.5 and 1.0 wt% MVR which varies between 3.394 and 7.793 mm^2^·h^−1^ for the MVR/VAC-HEMA0.5 system and between 1.348 and 4.130 mm^2^·h^−1^ for the MVR/VAC-HEMA1.0 system, depending on the pH medium. On the other hand, this parameter is less rapid in the case of the two other systems containing 1.5 and 2.0 wt% MVR with diffusion coefficients varying between 0.788 and 1.961 mm^2^·h^−1^ for the MVR/VAC-HEMA1.5 system and between 0.629 and 1.298 mm^2^·h^−1^ for the MVR/VAC-HEMA system.

### 3.3. Effect of pH Medium

**Figure 14:**
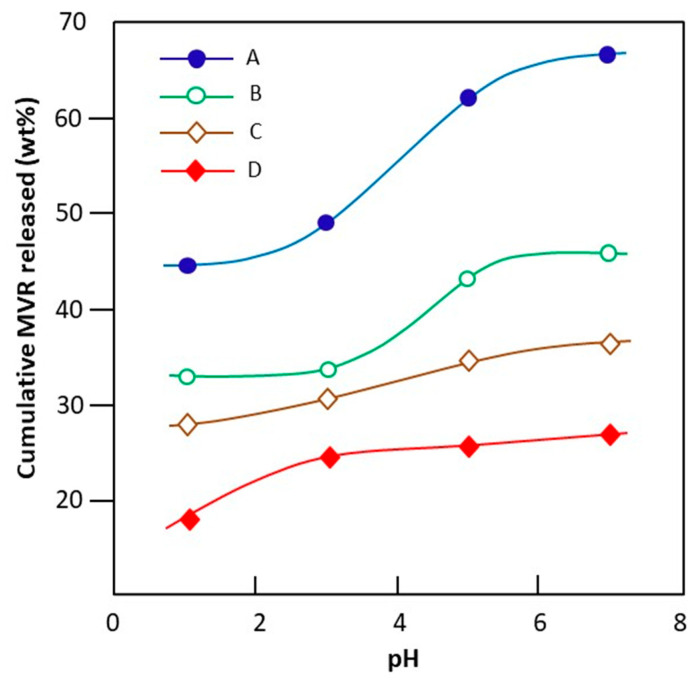
The change in the cumulative MVR released from MVR/VAC-HEMA0.5 (A), MVR/VAC-HEMA1.0 (B), MVR/VAC-HEMA1.5 (C) and MVR/VAC-HEMA2.0 (D) systems versus the pH of the medium taken at 3 days of the release time.

The effect of the pH medium on the release dynamic of MVR from the MVR/VAC-HEMA systems was studied over a period of 3 days of the release process and the results obtained are presented out in Figure 14. These curves reveal an increase in the percentage of the cumulative MVR released with the pH of medium, passing through an acceleration step between pH 3 and 5, which increased with the amount of MVR loaded except that of the drug carrier system containing the maximum amount of MVR (2.0 wt%) which stabilized in this pH range.

As stated earlier in Section 3.1.6, the swelling capacity of the VAC-HEMA carrier is virtually unaffected by the pH of the absorbed medium. In this case, the variation in the percentage of MVR released depends mainly on the interactions between pH and its solubility in the medium where it will be released. Indeed, the solubility of MVR in pH media 1 and 7 obtained in this work (Section 3.1.8) was found equal to 0.1313 and 0.1407 mg·mL^−1^, respectively. This seems to explain why the release of MVR increased with the pH of medium increased from 1 to 7.

The presence of MVR in the VAC-HEMA matrix made it possible to reduce the percentage of MVR released in media pH acid. This can be explained by a reduction in the swelling capacity of the MVR/VAC-HEMA system due to a rearrangement of the inter-chain hydrogen bonds of the hydroxyl-hydroxyl and hydroxyl-carbonyl type in this medium, thus reducing the affinity between VAC-HEMA and water molecules in favor of that between MVR and the copolymer. In this situation, fewer water molecules penetrate the copolymer matrix due to the hydrophobicity of the MVR physically linked to this material, resulting in less solvation of the drug and consequently less release in this environment.

### 3.4. Performance of the MVR/VAC-HEMA Drug Carrier System

The study of the effectiveness of the MVR/VAC-HEMA drug carrier system in the delivery of MVR in the different PH media investigated was carried out in this work on the basis of three essential factors: (i) the cumulative amount of MVR released, (ii) the stability of the release rate and iii) the duration of the release process. In general, the most effective system is the one that releases the greatest amount of drug in a neutral pH medium (intestinal) and little in an acidic pH medium (stomach) with a uniform release rate and for a long period. The profile of each kinetic curve in Figure 10 reveals three main pseudo-linear steps, the durations of which depend on the pH of the medium and the amount of drug initially loaded into the VAC-HEMA carrier.

The percentage of MVR released and its instantaneous release velocity for each period and each system are deduced from the coordinates and slope of each corresponding pseudo-linear curve, respectively, and the results obtained are gathered for comparison in Table 4. The first step, which is the fastest and the shortest, occurs during the first 2 to 5 h of the release process is characterized by a significant release percent of MVR in these different media (8.0–37.7 wt%) with a stable release rate mainly of 6.0 wt%∙h^−1^. The second stage, which comes just after, is medium and goes from 8 to 31 h of the release process. During this period, between 6.0 and 26.5 wt% of MVR were uniformly released with a release rate varying between 0.34 and 1.54 wt%∙h^−1^, depending on the pH medium and the MVR initially loaded in the VAC-HEMA carrier. The third stage of the release process is the longest (36 to 59 h) during which a small percent of MVR are released (0.3–8 wt%) with a stable release rate oscillating between 0.10 and 0.72 wt%∙h^−1^, depending on the pH of the medium and the MVR initially loaded in the carrier. The performance of the MVR/VAC-HEMA drug carrier systems was estimated according to the criteria cited above which are: (i) an adequate fraction of drug released in the intestinal transit (neutral pH medium), (ii) a minimum fraction of drug released in the stomach (acidic pH medium) and iii) a stability in the drug release rate over a long period of time. Considering these criteria, it was found that the MVR/VAC-HEMA systems containing 0.5 and 1.0 wt% MVR contents seemed to give the best performances with 62.2 and 45.0 wt% of this drug released in neutral pH medium and 36 and 28.3% by weight in the acidic media (pH 1 and 3), respectively.

According to the literature [34], it was found that the mean gastrointestinal transit time (GITT) which depends on the age, sex and weight of the person is between 53 and 88 h distributed over three main steps: (i) gastric transition time between 1 and 4 h (pH 1.5–3.5), (ii) intestinal transit time between 4 and 12 h (pH 7–9) and (iii) colon transit time between 48 and 72 h (pH 5–7 h). Taking into account the release of the MVR in different pH media of Table 4 and the GITT distribution on the different digestive organs approached by Beltzer et al. [34], it was possible to approximately estimate the percentages of cumulative MVR released from the MVR/VAC-HEMA drug carrier systems in different organs. The average stomach/digestive organs rate (*SDO*) was evaluated, disregarding the effects of microorganisms and enzymes, using Equation (8) and the data obtained are illustrated in Table 5.

(8) $$SDO\left(wt\%\right)=\frac{r_{St}}{r_{st}+r_{sint}+r_{col}}\times 100,\;$$

where $r_{st}$, $r_{sint}$ and $r_{col}$ are the MVK in wt% released in the stomach, small intestine and colon, respectively, during a certain transit time.

For the short gastro intestinal transit, these data reveal that the MVR/VAC-HEMA drug system delivers in the stomach between 8.76 and 14.38 wt% of the initial MVR loaded and between 14.81 and 19.00 wt% for the long gastro intestinal transit. The systems containing 0.5 and 1.0 wt% of this medication show the best performance because they were able to reduce the minimum percentage release of MVR in the stomach to 8.76 and 14.38 wt% for MVR/VAC-HEMA0.5 and MVR/VAC-HEMA1.0, respectively. This is beneficial because among the goals in the drug delivery field is their delivery in the intestines, which are the main seat of absorption and not the stomach which is the seat of the chemical and mechanical crushing of food. Sometimes, when a drug is administered directly orally, a considerable amount will be destroyed in the stomach under the effect of enzymes and the acidity of the environment and therefore only a part of the drug will reach its objective (the intestines). In addition, the products resulting from the breakdown of this drug in the stomach can cause several side effects. In this case, the smaller the amount of drug released into the stomach, the more these side effects are reduced. On the other hand, the incorporation of medication in the copolymer used as carrier such as VAC-HEMA also allows the reduction in the prescribed dose for the patient, and this is performed by subtracting a destroyed part of the drug in the stomach. The dosage of MVR prescribed for a patient according to sex, age, weight and health can be easily controlled by the amount of MVR/VAC-HEMA drug carrier system administered.

## 4. Conclusions

The objectives of this work were successfully achieved. Indeed, the photo-copolymerization of 2-hydroxyethyl methyl methacrylate with vinyl acetate in a desired percentage (~15 wt% vinyl acetate) in the presence of desired ratios of AVR was successfully performed under LED light. The uniform dispersion of the Mevacor particles at their molecular level in the VAC-HEMA matrix, confirming the obtaining of a solid solution, has been demonstrated by the DSC and XRD and SEM analysis methods. The swelling investigation of VAC-HEMA material in pH medium ranged between 1 and 7 demonstrated that the swellability of this material was 45.20 ± 0.82 wt% which is practically unaffected by the pH of the medium. No significant cytotoxicity of virgin VAC-HEMA and MVR/VAC-HEMA drug carrier systems was revealed by the LDH test and the percentage of the cell adhesion on these systems examined by MTT assay indicated much higher than the negative control. The “in vitro” of the Mevacor released from these systems revealed a maximum increase of 22- and 37-fold that dissolved as powder under the same temperature and media. These data also indicate that the highest Mevacor release occurs when the drug carrier system initially contained 0.5% MVR. In any pH medium, the incorporation of MVR into the copolymer matrix drastically decreased the percentage of the drug released. It was also found from these results that the cumulative MVR released increased with the pH medium. The MVR/VAC-HEMA drug delivery system containing 0.5 and 1.0 wt% MVR appears to provide the best performance with 62.2 and 45.0 wt% of this drug evenly released in neutral pH medium, respectively, and 36 and 28.3 wt% in pH medium 1, respectively.

Utilizing the Belzer approach, the results of average stomach/digestive organs rate indicated that the MVR/VAC-HEMA drug carrier system delivers between 8.76 and 14.38 wt% of Mevacor in the stomach during the short GITT and between 14.81 and 19.00 wt% during the long GITT. The drug carrier systems including 0.5 and 1.0 wt% MVR demonstrate the highest performance, reducing the minimum release of this medication in the stomach to 8.76 and 14.38 wt%, respectively. This is advantageous, because one of the intended targets in the field of drug delivery is their release directly into the intestines, which is the main site of food and drug absorption.

## Figures and Tables

**Table 1:** Kinetics parameters deducted from the straight lines of the curves $Ln\left(m_{t}/m_{\infty }\right)$ of virgin VAC-HEMA hydrogel versus *Ln*(*t*) at 37 °C obtained in different pH media.

	VAC-HEMA Hydrogel			
pH Medium	1	3	5	7
*n*	0.488 ± 0.087	0.492 ± 0.086	0.502 ± 0.073	0.493 ± 0.072
*k*(h)^−1^	0.222 ± 0.120	0.256 ± 0.111	0.243 ± 0.121	0.289 ± 0.118
*D*·10^−3^(mm^2^·h^−1^)	1.901 ± 0.054	2.273 ± 0.034	2.435 ± 0.028	2.792 ± 0.025

**Table 2:** Comparison between the maximum solubility (mg∙mL^−1^) of MVR released from the MVR/VAC-HEMA system with that directly dissolved as powder at 37 °C in pH media 1 and 7.

	MVR (mg·mL^−1^)	
pH	1	7
Powder	0.0060 ± 0.0013	0.0038 ± 0.0015
MVR/VAC-HEMA1.5	0.1313 ± 0.0010	0.1407 ± 0.0012

**Table 3:** *k′* and *D′* values deducted from the kinetic curves indicating the variation in $(m_{t}/m_{0})\;$ versus the square root of time.

System	pH	*k′*·10^2^ (h^−0.5^)	*R^2^*	*D′*·10^−3^ (mm^2^·h^−1^)	System	*k′*·10^2^ (h^−0.5^)	*R^2^*	*D′*·10^−3^ (mm^2^·h^−1^)
I	1	3.29	0.9966	3.394	III	2.00	0.9900	0.788
	3	3.58	0.9454	4.473		2.59	0.9969	1.979
	5	6.11	0.9874	9.221		2.64	0.9961	1.741
	7	5.57	0.9818	7.793		2.81	0.9881	1.961
II	1	2.11	0.9883	1.348	IV	1.46	0.9839	0.629
	3	2.79	0.9844	2.203		1.85	0.9785	0.984
	5	3.65	0.9952	3.850		2.05	0.9909	1.266
	7	3.79	0.9898	4.130		2.17	0.9913	1.298

I: MVR/VAC-HEMA0.5; II: MVR/VAC-HEMA1.0; III: MVR/VAC-HEMA1.5; IV: MVR/VAC-HEMA2.0.

**Table 4:** Distribution of the cumulative MVR released from the MVR/VAC-HEMA on the different pseudo stable zones.

System	pH	Pseudo- Stable Zone (h)	Cumulative MVR Released (wt%)	Release Rate (wt%/h)	System	Pseudo- Stable Zone (h)	Cumulative MVR Released (wt%)	Release Rate (wt%/h)
I	1	0–3	22.0 ± 0.3	7.33 ± 0.10	III	0–3	18.0 ± 0.5	06.0 ± 0.17
		5–36	12.0 ± 0.3	0.41 ± 0.01		7–36	10.0 ± 0.4	0.34 ± 0.01
		36–72	02.0 ± 0.2	0.07 ± 0.01		36–72	0.3 ± 0.2	0.01 ± 0.01
	3	0–5	24.0 ± 0.3	4.80 ± 0.06		0–3	18.0 ± 0.5	06.0 ± 0.17
		5–36	26.5 ± 0.6	0.85 ± 0.02		3–35	15.0 ± 0.5	0.47 ± 0.02
		36–72	06.5 ± 0.3	0.18 ± 0.01		36–72	01.0 ± 0.2	0.03 ± 0.01
	5	0–5	32.0 ± 0.4	6.40 ± 0.08		0–3	19.0 ± 0.5	6.33 ± 0.17
		5–22	25.0 ± 0.3	1.47 ± 0.02		3–16	13.0 ± 0.4	01.0 ± 0.03
		26–72	08.0 ± 0.2	0.17 ± 0.01		16–72	06.0 ± 0.3	0.11 ± 0.01
	7	0–5	32.7 ± 0.5	6.54 ± 0.10		0–3	19.0 ± 0.6	6.33 ± 0.02
		5–26	22.5 ± 0.3	1.07 ± 0.01		3–16	20.0 ± 0.6	1.54 ± 0.05
		26–72	07.0 ± 0.3	0.15 ± 0.01		16–72	06.0 ± 0.4	0.11 ± 0.01
II	1	0–2	10.0 ± 0.3	5.00 ± 0.15	IV	0–2	05.0 ± 0.2	2.50 ± 0.10
		2–12	11.3 ± 0.3	1.13 ± 0.03		2–10	08.0 ± 0.3	1.00 ± 0.04
		17–72	4.0 ± 0.2	0.72 ± 0.01		10–72	08.0 ± 0.4	0.13 ± 0.01
	3	0–2	11.0 ± 0.4	5.50 ± 0.20		0–2	08.0 ± 0.4	4.00 ± 0.20
		2–14	15.0 ± 0.5	1.25 ± 0.04		2–16	10.0 ± 0.4	0.71 ±0.03
		14–72	3.0 ± 0.2	0.05 ± 0.01		16–72	06.0 ± 0.4	0.11 ± 0.01
	5	0–2	13.0 ± 0.3	06.5 ± 0.15		0–2	09.0 ± 0.2	4.50 ± 0.10
		2–17	15.0 ± 0.4	01.0 ± 0.03		2–11	09.0 ± 0.4	1.00 ± 0.04
		19–72	7.0 ± 0.3	0.13 ± 0.01		13–72	06.0 ± 0.3	0.10 ± 0.01
	7	0–2	14.0 ± 0.2	07.0 ± 0.10		0–2	10.0 ± 0.2	5.00 ± 0.10
		2–19	6.0 ± 0.4	0.35 ± 0.02		2–13	11.5 ± 0.3	1.06 ± 0.03
		19–72	6.0 ± 0.3	0.11 ± 0.01		13–72	06.5 ± 0.3	0.11 ± 0.01

I: MVR/VAC-HEMA0.5; II: MVR/VAC-HEMA1.0; III: MVR/VAC-HEMA1.5; IV: MVR/VAC-HEMA2.0.

**Table 5:** Distribution of the total MVR released from MVR/VAC-HEMA drug carrier systems on the principal digestive organs timed according to the Belzer approach.

MVR/VAC-HEMA System	Stomach Transit (wt%)		Small Intestine Transit (wt%)		Colon Transit (wt%)		SDO (wt%)	
Transit Time	Min (1 h)	Max (4 h)	Min (4 h)	Max (12 h)	Min (48 h)	Max (72)	Min (48 h)	Max (72)
MVR/VAC-HEMA0.5	6.07	13.37	26.16	40.19	33.06	36.66	10.86	14.81
MVR/VAC-HEMA1.0	6.00	18.41	20.00	28.10	42.52	45.82	8.76	19.90
MVR/VAC-HEMA1.5	5.25	10.13	14.70	17.50	23.19	25.83	12.17	18.95
MVR/VAC-HEMA2.0	6.25	8.21	12.12	20.60	25.35	28.00	14.38	14.45

## Data Availability

Not applicable.
